# P19 Impact of the introduction of the EUCAST 2020 susceptibility interpretation rules for *Pseudomonas aeruginosa* on selection of antibiotic therapy

**DOI:** 10.1093/jacamr/dlae136.023

**Published:** 2024-08-23

**Authors:** Chai Feng Benjamin Chan, Jumoke Sule, Theodore Gouliouris

**Affiliations:** School of Clinical Medicine, University of Cambridge, Cambridge, UK; Clinical Microbiology and Public Health Laboratory, Cambridge University Hospitals NHS Foundation Trust, Cambridge, UK; Clinical Microbiology and Public Health Laboratory, Cambridge University Hospitals NHS Foundation Trust, Cambridge, UK; Department of Medicine, University of Cambridge, Cambridge, UK

## Abstract

**Background:**

In 2020, EUCAST changed the interpretation of WT *Pseudomonas aeruginosa* susceptibility to the first-line agents ciprofloxacin, piperacillin/tazobactam and ceftazidime from susceptible (S) to susceptible, increased exposure (I). The breakpoint changes were implemented at Cambridge University Hospitals (CUH) in March 2020, initially with no explanatory notes, and from June 2020 with the addition of an interpretative comment.

**Objectives:**

To study the impact of implementing the EUCAST susceptibility interpretation of WT *P. aeruginosa* on carbapenem prescribing at CUH.

**Methods:**

In this service evaluation, we analysed all clinical isolates of *P. aeruginosa* between 1 January 2020 and 30 September 2020. Only WT (fully susceptible isolates) were included. We retrospectively collected epidemiological data, clinical background, treatment information and Microbiology involvement and analysed this data to identify prescription rates and factors influencing meropenem prescription in *P. aeruginosa* infections, comparing three periods: Period 1 (pre-transition), Period 2 (transition) and Period 3 (post-transition).

**Results:**

In total, 104 patients were included in the study. Meropenem use across all three periods was similar (17.6%, 18.4% and 12.5%, *P*=.799). Selective susceptibility reporting of meropenem susceptibility differed between periods, with a transient increase in reporting during period 2 (11.8%, 36.1% and 6.3%, respectively [*P*=.004]). Reporting of susceptibility to meropenem was associated with increased odds of meropenem prescribing (OR 4.3, 95% CI 1.40–13.7, *P*=.011).

**Conclusions:**

The changes to the EUCAST breakpoints for *P. aeruginosa* did not have a significant impact on carbapenem prescription at CUH. This in part could be explained by selective susceptibility reporting of results.

